# Where Do Ethno-Linguistic Groups Meet? How Copresence during Free-Time Is Related to Copresence at Home and at Work

**DOI:** 10.1371/journal.pone.0126093

**Published:** 2015-05-21

**Authors:** Ott Toomet, Siiri Silm, Erki Saluveer, Rein Ahas, Tiit Tammaru

**Affiliations:** 1 Department of Economics, Tartu University, Tartu, Estonia; 2 Department of Geography, Tartu University, Tartu, Estonia; 3 Positium LBS, Tartu, Estonia; 4 Faculty of Architecture and the Built Environment, Delft University of Technology, Delft, The Netherlands; Centre for Ecological and Evolutionary Studies, NETHERLANDS

## Abstract

This paper analyzes ethnic segregation across the whole activity space—at place of residence, place of work, and during free-time. We focus on interethnic meeting potential during free-time, measured as copresence, and its relationship to copresence at place of residence and work. The study is based on cellphone data for a medium-sized linguistically divided European city (Tallinn, Estonia), where the Estonian majority and mainly Russian-speaking minority populations are of roughly equal size. The results show that both places of residence and work are segregated, while other activities occur in a far more integrated environment. Copresence during free-time is positively associated with copresence at place of residence and work, however, the relationship is very weak.

## Introduction

Segregation research is typically focused on places of residence. This is because common datasets typically only include information on where people live, resulting in what is sometimes called the analysis of “sleeping population” [1]. In contrast, the activity-based approach in segregation studies relates the spatial routines of people not just to their homes but to their other meaningful places as well [2, 3]. This approach distinguishes three main activity types: those related to home, work, and free–time activities [4]. Free time, or time spent out of work and family obligations, is sometimes also denoted as leisure time or “out-of-home non-employment activities”, to stress that this includes both leisure and maintenance activities [5]. Further, research shows that the share of daily hours devoted to leisure time has significantly increased in modern societies, and that these three activities cluster in space in different ways [6].

We build our study on the emerging branch in the segregation research that goes beyond residential neighborhoods. The previous papers, analyzing more than just one activity place, typically focus on pairwise relationship between these, often between home and work [7, 8]. The innovative aspect of our study is to compare levels of ethnic segregation across the whole activity space — home, work, but also other places such as free–time places —, and to incorporate a new dimension — time — into the typically only space-based segregation studies. We measure segregation through copresence, i.e. whether members of different ethnic groups are in the same place at the same time. More specifically, this paper analyzes how the level of segregation compares at these three activity sites, and how ethnic copresence at places of residence and work is related to that of outside of home and work.

We draw our empirical evidence from a racially homogeneous but ethno-linguistically divided European city (Tallinn, Estonia) of about 400 000 inhabitants, where Estonian-speaking majority and Russian-speaking minority are of an almost equal share of total population. We rely on a novel mobile positioning dataset where we observe the space-time activity pattern of cellphone users. It allows us to include both space and time dimension into our analysis. Studies based on mobile phone data are rapidly making their way into social science research, providing new and exciting evidence on how ethnic and racial segregation is produced and reproduced in contemporary cities [9]. The dataset we use allows us to measure whether members of different ethno-linguistic groups have been in the same part of the city at the same time and accordingly had a possibility to meet and interact with each other. Our analysis focuses on the exposure dimension of segregation, measured through the homophily index [10, 11]. This allows us to compare levels of ethnic segregation at places of residence, work and free–time. Thereafter, we quantify by regression analysis the extent by which copresence at place of residence and work are related to copresence during free–time.

## Theoretical background

### Patterns of Segregation at Home, Work and during free–time

There is a large body of literature about residential segregation both in the U.S. and Western Europe (see [1, 12] for a recent review). It shows that people of similar ethnic or racial background tend to congregate, forming segregated, homogeneous and ethnoracially distinct neighborhoods. Although more-and-more evidence suggests that urban neighborhoods both in the U.S. and Western Europe are becoming increasingly diverse [13, 14], residential segregation has remained a serious concern. First, despite declining trends, levels of it are still high, social networks remain detached, and social interaction between ethnic groups limited [12, 15]. Second, not all ethnic and racial groups experience increasingly diverse neighborhoods. In the U.S., residential segregation is still very high between Whites and African Americans, although the trend is downward [16]. This has recently led to an increasing number of studies about the extent, by which the patterns of residential segregation are reflected in workplace, where social interaction between ethnic and racial groups is potentially more intense [7].

Studies of workplace segregation show that employment is inherently spatial — minority workers tend to be concentrated not only in certain jobs and industries, but also in certain areas within the city [17, 18]. Patterns of residential and workplace segregation are related for different reasons, such as neighborhood–based ethnic networks and job referrals, location of ethnic enterprises in corresponding residential areas, and spatial limits to commuting. However, studies both in the U.S. and in European context [7, 8] show that levels of workplace segregation are lower compared to levels of residential segregation. Factors, such as employers’ need for workers with different skills, a higher dispersal of jobs suitable for immigrants in the city, and regulations promoting equal opportunity at workplaces, contribute to lowering the level of workplace segregation [18].

In addition to geographies of home and work, there is an increasing interest in understanding ethnic and racial segregation in other places [12]. Activities related to home and work are often spatially restricted and form a routine activity space [19], whereas there is more freedom to choose with whom and where to spend the free–time [20]. Thus, free–time activities may give us important insight into the potential for ethnic and racial interaction. The previous studies, analyzing ethnic differences in spatial mobility outside home and work, have typically focused on a single type of activities, such as attending church services [21], going to casinos [22], or visiting national parks [23]. There are fewer studies that capture the complete out-of-home non-employemnt activity pattern.

From the theoretical viewpoint, the potential for inter-ethnic contact during free–time is ambiguous. On the one hand, there is evidence that different ethnic groups often spend their free–time in separate places [24, 25] performing different activities [23, 26]. For example, the pattern of attending churches [21] and visiting urban parks [27] differ significantly by ethnic and racial groups. On the other hand, common-interest–based activities, such as sporting or attending popular cultural and other events, can trigger people out of their networks, and facilitate inter-group contact and integration [28, 29]. In other words, various activities performed outside home and work can integrate people on basis of their common interests irrespective of ethnic background [30].

To sum up, studies of ethnic and racial segregation patterns are mainly focused on a single place in the activity space, and more recently also on pairwise links between these, typically between home and work. The motivation of this paper is to understand how segregation and integration processes operate across the full activity space, anchored not only at home and at work but also at other places where people spend their time. We aim to capture more detailed space-time patterns of ethnic and racial groups in contemporary cities. The very first studies on segregation across the full activity space have emerged only recently [9, 31], and we contribute to them by (a) measuring explicitly levels of segregation at home, at work and in other places; and (b) analyzing how the latter is related to segregation at places of residence and work.

### Explanations of free–time Segregation

The two common explanations of ethnic and racial segregation are related to differences in the socio-economic status and discrimination, and to homophilic preferences — a tendency of similar people to group together [15]. The first of these explanations attributes ethnic and racial segregation to different degrees of disadvantage in social status and material wellbeing. The low level of material wellbeing among minorities is caused by their more disadvantaged position in the labor market. As a consequence, they tend to be over-represented in unstable and low-productivity jobs [32]. This leads into differences in free–time use through three channels: through income, through networks, and through status identification. Low income limits the possibilities to participate in a large number of out-of-home activities [33]. In this way, being financially constrained in leisure time activities becomes part of the marginalization process as well.

Workplace is also an important sphere where social networks are formed [34, 35]. This is because people often spend their free–time with coworkers either in an organized way (e.g. employer–arranged events such as team-building initiatives) or by initiating joint free–time activities during more leisurely settings at work. All this implies that labor market segmentation and workplace segregation have important impact on free–time segregation as well [36]. Finally, people increasingly use leisure to stress their identity and status and free–time activities thus form an integral part of socialization and life-style, freedom to undertake desired leisure activities signals individual success [37, 38]. Leisure has therefore been characterized as the “the long arm of work” [39], an increasingly important part of the activity space that produces and reproduces ethnic and racial segregation in contemporary cities.

The preference–explanation argues that there is something more than just socio-economic disadvantage that causes individuals to spend their free–time with co-ethnics. Homophilic preferences often arise from a shared cultural background, such as origin, language, traditions, and socialization practices [23, 40]. The immigration process facilitates creation of co-ethnic networks that in turn lead to ethnic neighborhoods and workplaces [41]. Previous research shows that people tend to spend their free–time together with representatives of their own group, rather than with members of other ethnoracial groups [21, 24]. Further, ethnic and racial groups often choose to undertake specific activities at specific locations in order to stress their difference from the others and to maintain their identity and heritage. free–time activities may thus play an important role not just in integration but in preserving the identity of minorities as well [42].

In summary, both of these explanations argue that own-group members are over-represented in individual networks. The existing evidence suggests that the level of workplace segregation is lower than that of residential segregation. However, according to our knowledge, explicit comparisons of segregation across the whole daily activity space — home, work and other places — are still missing. The explanations above suggest that free–time may be both less and more segregated than place of residence and work. Less advantaged position of minorities as well as own-group preferences facilitate spending free–time together with co-ethnics. Even more, because distance matters, and our daily activities are centered around the location of home and workplace, both residential and workplace segregation are mirrored in leisure time segregation. The social and ethnic networks arise both in residential neighborhoods and at workplaces, and influence segregation during free–time as well.

## Data

In this paper the mobile phone data management and analyses were strictly following all data security and privacy requirements, as specified by the European Parliament in the Data Protection Directive (Directive 1995/46/EC) and the General Data Protection Regulation, the Electronic Privacy Directive (Directive 2002/58/EC), the Data Retention Directive 4 (Directive 2006/24/EC). The use of Estonian passive mobile phone data for scientific research concerning ethical and privacy issues was approved by The Estonian State Data Protection Agency. The anonymity of all respondents is strictly protected.

### Study Area

Our study is based on Tallinn, the capital of Estonia. We analyze the segregation of two ethno-linguistic groups, Estonian-speakers and Russian-speakers. The Russian-speakers currently account for 29 per cent of the population in the country. This group formed mainly during the Soviet period (1944–1991) when a large number of immigrants arrived primarily from Russia (80% of minorities), Ukraine and Belarus [43]. 90% of them speak Russian as their mother tongue. A substantial number of immigrants moved to Tallinn where they currently form almost a half of the population despite of being minority country–wide. According to 2011 census, 55% of population in Tallinn identifies themselves as ethnic Estonians, 42% as either Russians, Belorussians or Ukrainians, and 3% have other ethnic background; country-wise, the corresponding figures are 69%, 29% and 2%. Already in the 1970s, the city was ethnolinguistically deeply divided along the languages, with both residential neighborhoods, workplaces and schools highly segregated [44, 45]. Ethnolinguistic composition of the city has not changed much since then despite the immigration ceasing after the collapse of Soviet Union in 1991.

Tallinn is a very interesting city for our analysis for several reasons. First, the population of the city is almost equally divided between the Estonian and Russian ethno-linguistic groups. Second, the language groups are distributed rather unequally across the city despite their similar city-wide size. This is due to the sorting and selection mechanisms of the Soviet era, working in a different way than those in the Western cities [46]. Most importantly, ethnic minorities are living in the apartments constructed during the Soviet period because the central housing allocation system favored newly arriving residents, while Estonians are distributed more evenly [47]. Third, there has been almost no new immigration over the past 25 years. This allows us to follow segregation for a well-established community with a long history of co-residence.

### Passive Mobile Phone Positioning Data

We base our analysis on a unique cellphone usage data from the largest mobile service provider in Estonia, EMT. Approximately 96% of the adult population in the country use cellphones and EMT’s market share in Tallinn is 39% [48]. Strictly speaking, we analyze the behavior of EMT customers only. However, we believe that these results generalize to the whole population of Tallinn (see Discussion section below). The type of data we use is commonly referred to as “passive positioning data,” where “passive” refers to the fact that it is extracted from the memory files held by mobile operators. The passive mobile positioning database is based on Call Detail Records (CDR), where each CDR is described by the time and location of the call activities (calls, text messages and multimedia messages). Typically for the passive data, we do not observe the actual location but rather the Cell Global Identity (CGI), i.e. the network antenna which processed the outgoing call. This gives us a spatial resolution of a few hundred meters in the most dense urban environments.

The data include the start time of each call activity and the corresponding location. Every network user (as identified by a SIM card with a unique phone number) is assigned a random identification tag, making it possible to track the same user over time. The data also include some background information about the phone users (SIM card owners). The most crucial variable for this study is the preferred language. Since Estonia is an ethnolinguistically dividend country, it is common that businesses in the service sector collect such information. Preferred language thus serves as a proxy for ethnic background as virtually all of those who identify themselves as ethnic Estonian also list Estonian as their first language, while most of those from other ethnic backgrounds mainly use Russian [49]. The database also includes information on gender and age. In addition to these three variables, we calculate the place of residence and a place of work to each cellphone owner based on timing, location and regularity of the calls using the methodology by Ahas et al [50]. The home and workplace are determined as the most frequently used anchor points (network antennas) based on the chronological variability of the calls. Normally, users have one home and one work-time anchor point but in some cases they may also have two home or two work-time anchors. The algorithm takes into account the potential switching of antennas by aggregating the neighboring parallel anchor points. The method works well for frequent cellphone users, for individuals with low number of calls the home and workplace may be unstable.

**Fig 1:**
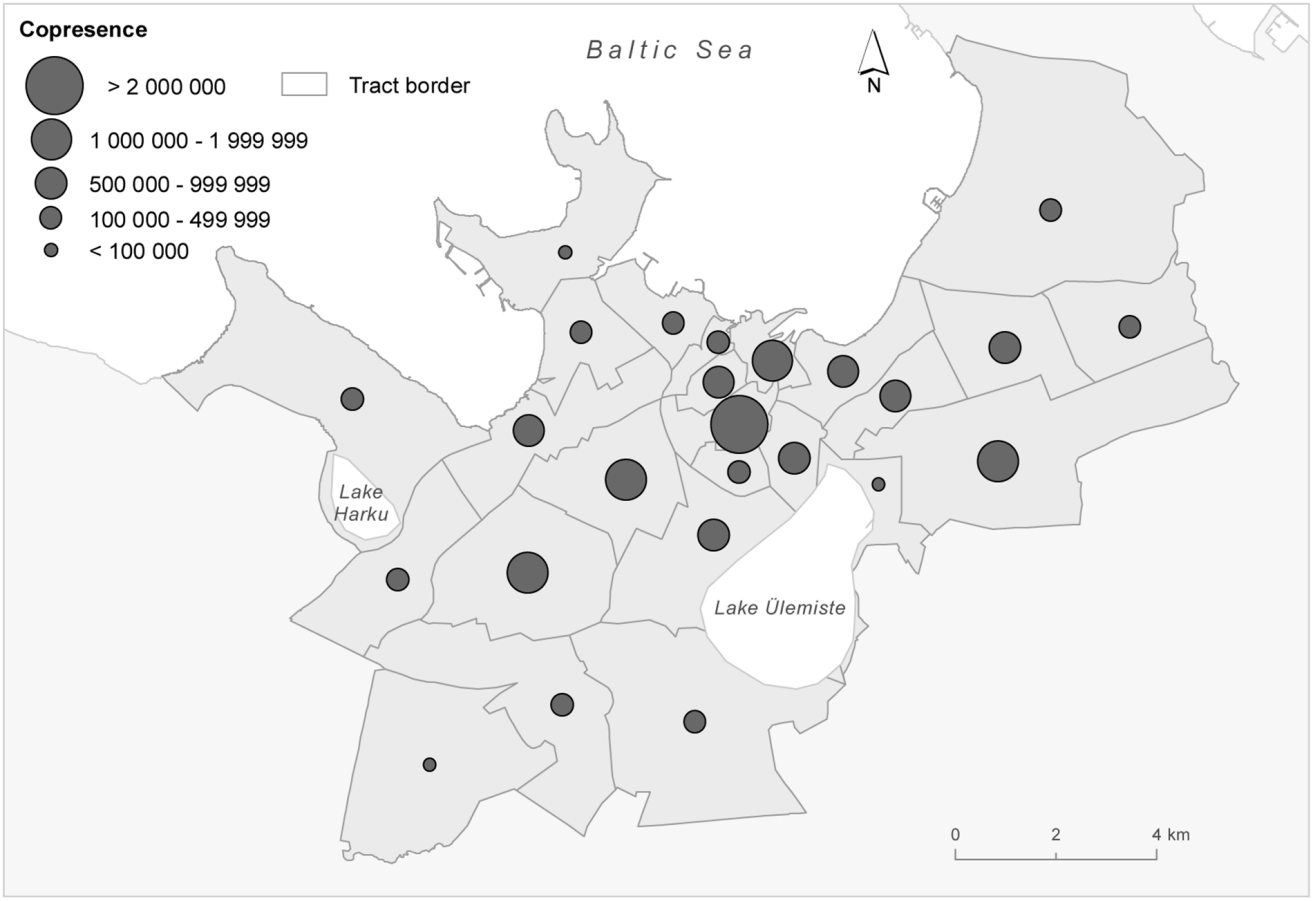
Estonian-Russian Copresence during free–time by city tract. The study area, Tallinn city, is shaded and tract boundaries are marked in dark gray. Copresence is measured in number of meetings in the sample over the observation period (∑_*j* < *i*_∑_*i*_ *p*_*ij*_ in the sense of expression Eq (1)).

The data cover the year 2009 and our sample contains individuals who are at least 18 years-old, and whose permanent place of residence in 2009 was in Tallinn based on our calculations. The sample is based on the distribution of Estonian-speaking and Russian-speaking residents in city tracts as in 2000 census. The tracts are defined based on the housing type and main roads to ensure their well-connected and relatively homogeneous structure. There are 25 tracts in Tallinn, see map in Fig 1. The sample includes 5,200 people, selected from the all available data with valid language and place-of-residence information in a way to preserve the census 2000 population proportions across the tracts and ethnic groups. The main factor determining the sample size was the low number of Russian-speakers with valid data in some smaller tracts. The number of calls are not significantly different between Estonian and Russian-speakers.

## Method

### Activity Space

Our point of departure is the framework of space-time path in the activity space, widely used in time geography since Hägerstrand [51]. Traditionally, three types of places/activity sites are distinguished in the daily activity space: those related to residence (home), those related to business (work), and free–time activities [4]. Our cellphone usage database does not provide us with readily available information about the actual activities. Instead, we use the location of neighborhoods of residence (denoted by *R* below) and work (*W*) to approximate the corresponding activities (Golledge and Stimson, 1997). We term all the other places “free–time places” (*F*). More accurately *F* describes places for “out-of-home non-employment activities”, including non-free activities, such as shopping or visiting a doctor (see [5]). In this way our method captures all three places as “places” in a geographical sense. In particular, this also applies to workplace — we do not analyze the establishment where one works but a geographical area where the work is conducted. In the literature it is also common to analyze segregation at locations, such as residence or work, without any explicit information about the activities and establishments (cf. [7]). In this paper we extend a similar approach to the free–time places.

When determining proximity in a certain place (*R*, *W* or *F*) within the activity space (operationalized as copresence, see below), we include all potential meetings (see below) of the individual-of-interest at this place with everyone else across the whole activity space. Hence we measure proximity between individuals at the place of interest — residential neighborhood, workplace neighborhood, or participating in free–time activities — to all other individuals in all activities. Note that for some people neighborhoods of residence and work overlap.

### Copresence

We operationalize proximity as copresence. For face-to-face interaction, the persons have to meet — they must be present in the same place at the same time. Copresence analytically measures the potential of people seeing each other and feeling their nearness, and the possibility of such an interaction between them [52]. We calculate copresence from the passive mobile positioning data in the following way. Based on call activity, we determine in which network cells the individual made calls during one-hour intervals (we count intervals as full hours from midnight) and hence was present. We refer to these space-time units as timeframes. In this way we assign a unique timeframe to every call made in the network. Next, based on the timeframes, we compute copresence. For each individual *i*, we denote the timeframe of their call *k* by *c*_*ik*_. We define the dyadic copresence *p*_*ij*_ for individuals *i* and *j* as the number of timeframes where both of these individuals are present. Formally,

$$\begin{array}{c} p_{ij}=\sum 𝟙(c_{jk}\in C_{i}), \end{array}$$ (1)

where 𝟙(⋅) is the indicator function and *C*_*i*_ is the set of all timeframes where the individual *i* made at least one call. We do not distinguish between making one or more calls in a given timeframe because we are interested in presence, not in communication activity. In this way copresence describes the “nearness” of two individuals both in space and time; high level of copresence requires that the individuals are repeatedly near each other in different timeframes. Obviously, copresence does not capture the actual interaction; it is only a necessary condition for it. We refer to it as “meeting potential” or “potential to encounter” (see Wong and Shaw [31]). Our timeframes are probably too large for copresence to possess much predictive power for existence of the actual social ties. However, it still provides evidence for the potential of face-to-face meetings. Note that such approach is common in studies of residential segregation as well: one typically analyses the proximity in terms of residential location, the information about actual social interactions is rarely available. Analogously, a number of activity-space based segregation studies do not analyze proximity but only rely on overlap of activity spaces [31, 53, 54]. Finally, although we compute copresence based on network cells, we define the activity space places *R* and *W* based on city tracts. This is in order to smooth out the random errors resulting from cellphones occasionally connecting to different nearby antennas even if physically at the same place. Accordingly, free–time places *F* contain all the tracts that are neither residence nor work tracts and hence all the activities “elsewhere”.

The most important limitations of our copresence measure are the following. Intuitively, because we only observe the locations of cellphone calls, copresence is a sufficient, but not a necessary condition for being in a given timeframe. Another point to note is that copresence is based on the binary indicators for presence in a given timeframe. We do not take into account eventual presence in neighboring tracts and hence ignore the potential “across-the-border” meetings. However, this method is substantially simpler than potentially superior methods that weight space-time distance in a continuous way. Finally, we do not take into account the duration of stay in individual timeframes. For instance, individuals driving in the city may have copresence with many others despite of no chances to interact with them. Despite these limitations, our method is still an important step forward from census and register data based studies and allows to capture the meeting potential across the full activity space and at all times of the day.

### Measuring Exposure as Homophily

Our primary focus is the meeting potential between individuals from different ethnic groups. This is a measure of the exposure dimension of segregation [11]. We analyze segregation through homophily index, a version of the isolation index that is adapted for individual observations. We observe two types of copresence for the individual *i*: with those who speak the same language as *i* (*s*_*i*_), and with those who speak a different language (*d*_*i*_). The index measures the percentage of individuals’ own type copresence in their complete set of copresence. Hence the homophily for individual *i*, *h*_*i*_, can be written as:

$$\begin{array}{c} h_{i}=\frac{s_{i}}{s_{i}+d_{i}} \end{array}$$ (2)

Intuitively, homophily is the percentage of copresence with individuals who share the same language. In case of random meetings, the expected value of homophily equals the relative size of the individual’s own group in the population. As a relative measure, it is not affected by the pattern of cellphone usage across the activity space, as long as it is identical for both ethnic groups; however, similar homophily figures may mask widely different numbers of actual meetings.

The simplest interpretation of homophily assumes that the probability that social tie exists between individuals *i* and *j* is proportional to the corresponding pairwise copresence (meeting potential), and this probability is independent of language. This is a heroic assumption, but it is qualitatively similar to what is implicitly used when interpreting the residential or workplace segregation measures. Our interpretation still remains valid if this assumption is replaced by a more relaxed one that allows the likelihood of a social tie to differ for same- and different-language copresence.

We also calculate the dissimilarity index to capture the evenness dimension of segregation. The calculation of the index is based on “presence”, i.e. counting the number of timeframes with call activities by both Estonian-speaking and Russian-speaking individuals throughout the whole year by the city tracts.

### Quantifying the Relationship between Free–Time, Home and Work Homophily

Our main interest is related to the relationship between homophily in different places (*R*, *W* and *F*) of the daily activity space, in particular the association between *F*-homophily on the one hand, and *R*-homophily and *W*-homophily on the other hand. We start by presenting the homophily distributions across all three activity sites individually. Thereafter, we analyze the relationship between *F*-homophily, and *R*-homophily and *W*-homophily by multivariate regression where we also control for a number of other variables. Since the choice of place of residence and place of work may be influenced by the free–time environment, the regression does not necessarily determine the causal impact (see Ellis et al [7], for a related discussion). We estimate the following regression model:

$$\begin{array}{c} h_{i}^{F}=\alpha_{0}+{\bar{\alpha }}_{1}{\bar{h}}_{i}^{R}+\alpha_{1}\rho_{i}+{\bar{\alpha }}_{2}{\bar{h}}_{i}^{W}+\alpha_{2}\omega_{i}+\beta^{\prime }X_{i}+\epsilon_{i} \end{array}$$ (3)

Here, $h_{i}^{F}$ is the *F*-homophily of individual *i*; ${\bar{h}}_{i}^{R}$ and ${\bar{h}}_{i}^{W}$ are the average *R*-homophily and *W*-homophily in the residence- and work tract of individual *i*, and *ρ*_*i*_ and *ω*_*i*_ are corresponding individual deviations from that average. We choose to standardize the explanatory homophily measures (${\bar{h}}^{R}$, ${\bar{h}}^{W}$, *ρ*, and *ω*) while we express the dependent homophily measure *h* ^*F*^ in percent. This is because it is natural to understand the exposure to the other groups in terms of percentages (of copresence), while normalized explanatory variables give better idea about the differences across the city. The individual background variables ***X*** include age groups (< 20, 20–29, 30–54 and 55+), a gender dummy, and call activity groups (dummies for distribution quintiles). We regard the latter as a proxy for socioeconomic status. All models are estimated separately for both language groups.

## Results

### Patterns of Segregation at Home, Work and during free–time

We start with a pattern analysis of copresence and homophily in three main activity sites. We distinguish between copresence of Estonian-speakers (ET-ET), Russian-speakers (RU-RU) and between Estonian-speakers and Russian-speakers (ET-RU). Table 1 shows that 38% of ET–ET, 45% of ET–RU and 47% of RU–RU meetings occur in places of residence. Workplace accounts for slightly above 30% and free–time places roughly 20–30% of all encounters. Obviously, these figures are sensitive to the size of region (currently specified as city tract). In particular, choosing small home and work regions leads to a low share of *R* and *W*-copresence and a large share of *F*-copresence. The average homophily values (columns 5 and 6) range between 50%–65%, the exact order differs by group. As Estonian-speakers form a somewhat larger group in the population, we expect their homophily values to be slightly higher but this is only true for *F*-homophily. On average, Estonian-speakers appear to be more isolated in free–time activities, and less isolated at home. In contrast, Russian-speakers are most isolated at home and work with their homophily exceeding the value of that of the Estonian-speakers. In other words, it can be inferred that Russian-speakers are more inclined to cluster in coethnic residential neighborhoods and workplaces, but have a higher chance to meet Estonian-speakers during free–time activities. In general, these averages are comparable to the census proportion of 55% Estonians and 42% Russians. Hence the aggregate homophily is not radically different from what we would expect in case of the random meetings, although Russians experience notably more isolation at *R* and W.

**Table 1:** Percentage of copresence across different activity places and dyad types.

	Place by type (%)				Homophily (%) by place	
	ET-ET	ET-RU	RU-RU	All	ET	RU
*R*	38.1	44.9	47.1	43.5	50.2	65.5
*W*	32.9	30.5	34.1	32.4	56.1	65.2
*F*	29.0	24.6	18.8	24.1	58.3	54.4

**Fig 2:**
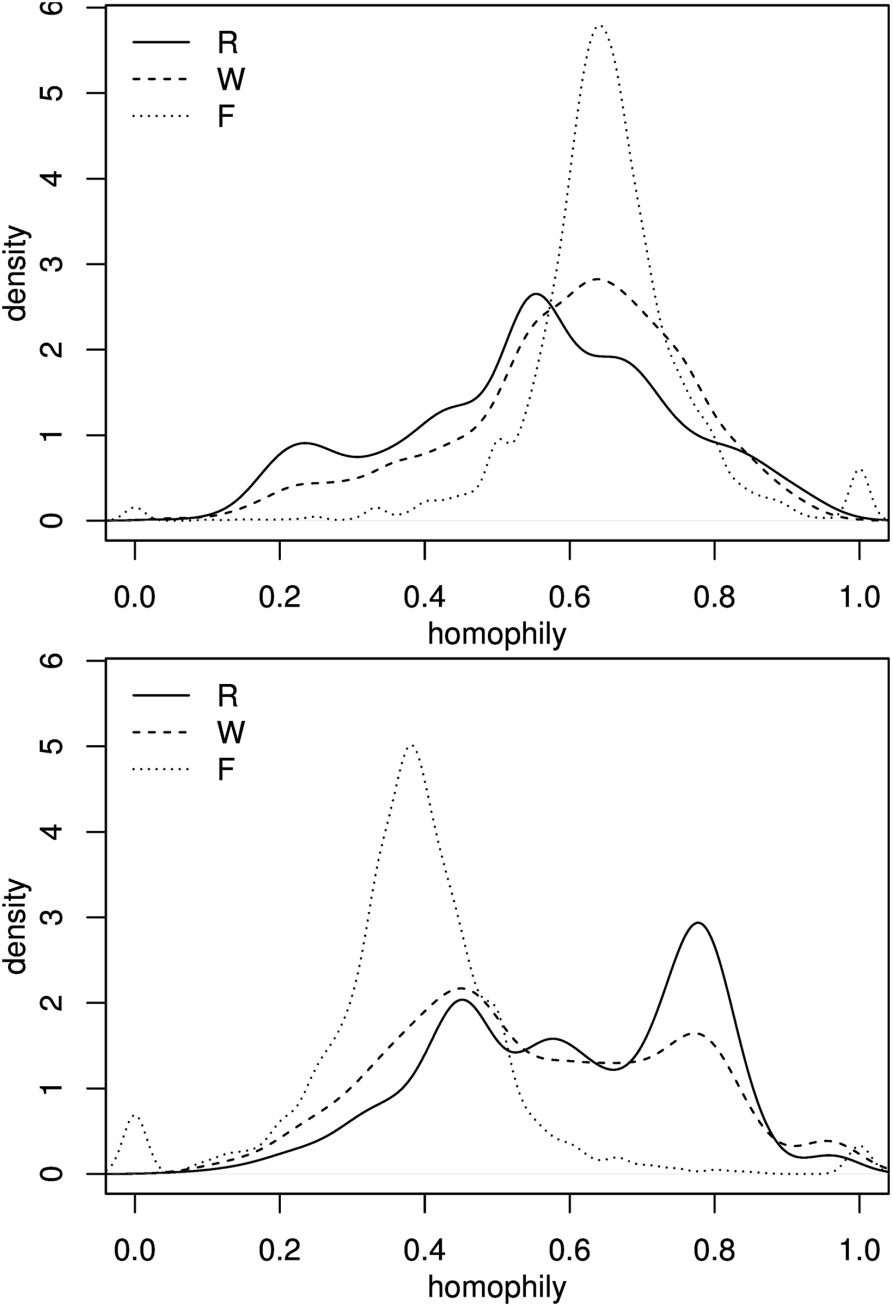
Kernel density estimates of the homophily distribution in *R*, *W* and *F*. (A) Estonian-speakers; (B) Russian-speakers.

The global homophily measures reported in Table 1 potentially mask important differences in the underlying distribution. The (marginal) homophily density in all three activity sites provides us a more detailed account of the segregation patterns (Fig 2). It appears that isolation at place of residence and place of work is distributed in a broadly similar way, and this is true for both language groups. Both across *R* and *W* tracts, homophily ranges roughly between 0.2 and 0.9, reflecting the population composition that varies across the city. The similarity of homophily distribution over *R* and *W* tracts is further highlighted by the rather similar values of the respective dissimilarity indices (*D* ^*R*^ = 0.41 and *D* ^*W*^ = 0.46). Fig 2 indicates that a number of Estonian-speakers live in tracts that are densely populated by Russian-speakers (where the homophily ranges between 0.2 and 0.3), while a significant fraction of Russian-speakers live in tracts that are more coethnic (homophily around 0.8). The *W*-homophily distribution of Russian-speakers has more mass at the less isolated end of the scale (homophily 0.4 and less).

In contrast, the *F*-homophily is distributed rather differently, with a prominent single peak in a much more narrow interval between 0.4 and 0.7, present for both groups, and corresponding approximately to the mean value in Table 1. Hence quite similar mean homophily indices in all three places (Table 1, columns 4 and 5) actually mask large differences in the corresponding homophily distributions. Fig 2 thus clearly indicates that both *R* and *W* tracts are moderately segregated while free–time activities take place in a much more integrated environment. In other words, when neither at work nor at home, the residents of Tallinn experience a significantly more ethnolinguistically mixed environment. The corresponding dissimilarity index (*D* ^*F*^ = 0.24) also indicates a more even distribution during free–time.

### The relationship between free–time segregation with residential and workplace segregation

Next we will perform a regression analysis with *F*-homophily being the dependent variable. We are mainly interested in the effect of *R*-homophily and *W*-homophily on *F*-homophily, both at the micro- and the macro-level (i.e., parameters *α*_1_ and *α*_2_ in regression Eq (3)). As explained above, we choose to measure *F*-homophily as a percentage, whereas both the *R*-homophily and the *W*-homophily are normalized. Hence the corresponding coefficients should be interpreted as the effect in percentage points per standard deviation of change. Results show that both home- and work-tract average segregation (${\bar{h}}^{R}$ and ${\bar{h}}^{W}$) are significantly and positively related to *F*-homophily in all three specifications (Table 2). The estimates for individual deviations from the tract averages, *ρ* and *ω*, are smaller and not significant for several models. All these estimates are fairly stable across the two model specifications. An increase by one standard deviation in *R*-homophily is related to an increase in *F*-homophily of no more than 3.2 percentage points (the coefficient for ${\bar{h}}^{R}$ for Estonian-speakers, Model 2). In other words, when both the *R*-homophily and *W*-homophily increase by one standard deviation, the *F*-homophily increases (not necessarily causally) by no more than 5 percentage points. In case of the average value for Estonian-speakers, this corresponds to experiencing free–time isolation index of 63 instead of 58%. Despite the high level of statistical confidence, this small change is probably not associated with substantially different experience in the free–time environment. In Model 2, we also introduce a number of additional explanatory variables in order to control for demographic and social composition of the population. All of these estimates remain small (although statistically significant in a number of cases), and do not exceed that of the most important explanatory homophily variable (${\bar{h}}^{R}$).

**Table 2:** Linear regression estimates. Dependent variable: *F*-homophily (in percent).

Model	1		2	
	ET	RU	ET	RU
constant	58.82***	53.42***	60.04***	52.91***
	*0.23*	*0.34*	*0.61*	*0.72*
*R*-homophily	2.93***	2.51***	3.18***	2.40***
	*0.23*	*0.30*	*0.25*	*0.32*
*ρ*	0.38**	0.46**	0.28*	0.36
	*0.15*	*0.23*	*0.17*	*0.24*
*W*-homophily	1.89***	1.92***	1.84***	1.91***
	*0.23*	*0.27*	*0.25*	*0.29*
*ω*	0.38***	0.48**	0.25*	0.14
	*0.14*	*0.24*	*0.15*	*0.25*
Female (Ref.)				
Male			−1.74***	0.53
			*0.43*	*0.45*
Age -20			2.08*	-1.46
			*1.21*	*0.94*
Age 20–30			1.34***	−0.27
			*0.49*	*0.67*
Age 30–54 (Ref.)				
Age 55-			−0.37	0.11
			*0.66*	*0.54*
Call quintile 1 (Ref.)				
Call quintile 2			−1.44*	0.83
			*0.75*	*0.71*
Call quintile 3			−0.54	0.43
			*0.66*	*0.68*
Call quintile 4			−1.23*	−0.14
			*0.68*	*0.75*
Call quintile 5			−1.49**	0.56
			*0.74*	*0.77*
# obs	2360	2016	1773	1489
*R* ^2^	0.1961	0.1527	0.2245	0.1467

Standard errors (in italics) are clustered across work and home tracts

Explanatory homophily measures (*R*-homophily, *ρ*, *W*-homophily and *ω*) are standardized.

*: *P* < 0.1,

**: *P* < 0.05,

***: *P* < 0.01

Fig 1 suggests that most of the interethnic meetings occur in the inner city. Tallinn, as a typical European city, is centered on a dense and vibrant downtown, which serves both as the central business district and as the main focal point for cultural activities and entertainment, irrespective of the language spoken. We analyze this further by partitioning the free–time copresence between “downtown” and “outskirts”. We present analogous regression outcomes for Model 1 in Table 3. The results clearly indicate that *F*-homophily in the downtown is unrelated to homophily both in the place of residence and work. All the estimates are small, and the models are not statistically significant (F-test *p* = 0.34 and 0.48 for Estonian and Russian-speakers respectively). This outcome is also stressed by a remarkably low dissimilarity index of 0.14 in the downtown. The opposite is true for the outskirts — both *R*-homophily and *W*-homophily are clearly related to *F*-homophily. Even more, the coefficient for ${\bar{h}}^{R}$ is substantially larger than that for the city as whole. The dissimilarity index, 0.28, is slightly larger as well. Hence our analysis suggests that the dense urban environment in the inner city more likely provides potential meeting places for ethnic groups than the outskirts. But even in the outskirts, the ethnic groups are substantially less segregated when not at home nor at work.

**Table 3:** Regression estimates for downtown and outskirts. Dependent variable: F-homophily (in percent).

	Downtown		Outskirts	
	ET	RU	ET	RU
*R*-homophily	0.43*	−0.14	4.22***	3.42***
	*0.23*	*0.27*	*0.32*	*0.35*
*ρ*	0.03	−0.49**	0.43**	0.38
	*0.23*	*0.24*	*0.19*	*0.25*
*W*-homophily	0.02	0.34	1.72***	1.59***
	*0.24*	*0.28*	*0.23*	*0.30*
*ω*	0.21	0.04	0.21	0.38
	*0.21*	*0.32*	*0.18*	*0.24*
# obs	2255	1914	2355	2015
*R* ^2^	0.002	0.002	0.229	0.167

Standard errors (in italics) are clustered across work and home tracts

Explanatory homophily measures (*R*-homophily, *ρ*, *W*-homophily and *ω*) are standardized.

*: *P* < 0.1,

**: *P* < 0.05,

***: *P* < 0.01

## Discussion and Conclusions

The analysis presented here provides a clear and unambiguous picture of the extent of ethnic segregation in Tallinn. While residential (home) and work locations are fairly segregated, places of other activities are not. For various reasons, people of different ethnic origin are living and working largely in separate neighborhoods. However, when they are neither at home nor at work, they have a better chance of meeting each other, typically in the city center. Moreover, mixing in those places is not conditional in any important way on the main sociodemographic characteristics. This outcome does not support the hypothesis that free–time activities are spatially segregated, suggesting instead that spatial segregation may not be a major problem in Tallinn. We cannot unambiguously say whether this is good or bad news. On the one hand, since Allport [55], a number of studies indicate that even superficial everyday meetings in public space may lessen the interethnic or interracial divide that hampers social capital and trust (see [56]). On the other hand, unpleasant encounters may breed defensiveness and frustration instead [57]. More research is needed here.

Our outcomes are not consistent with the results that workplace segregation is somewhat smaller compared to residential segregation [7, 8], although the difference in our data is small. We repeat here that we can only analyze segregation in space-time, not in actual activities. We also confirm a positive association between residential and workplace segregation on the one hand with free–time segregation on the other hand. The effects are small but larger than the effects of individual sociodemographic characteristics.

In this study we analyze exposure to population that is close both in space and in time. However, we have no information on the actual contacts and social ties of these individuals. This is a common problem in segregation research—for instance, the residential segregation analysis typically focuses on proximity in place of residence only. We extend the same approach to more spatial dimensions, and to time.

Ethnic and racial segregation during free–time can be explained by both socioeconomic disadvantages and by homophilic preferences. But what are the mechanisms which lead to free–time integration while both the residential and workplace neighborhoods remain segregated? A possible explanation may be related to city size and the role of the urban center. Although several neighborhood service centers exist in Tallinn, the ethnic geography of the free–time indicates that the strong and well-developed downtown provides a good mix of various amenities and services that potentially bring together different ethnolinguistic groups. This may not be true in more sprawled cities. Our results encourage stricter policies to counter urban sprawl and sociospatial fragmentation if more casual inter-ethnic contacts are deemed desirable. Urban sprawl has traditionally been considered a major problem mainly from the environmental perspective but possibly policies that strengthen city centers could also counter social fragmentation and increase the opportunities to meet others with different socioeconomic and ethnoracial backgrounds.

Our analysis is relying on data from a single cellular operator. To what extent can our results be generalized to the whole population of Tallinn? We argue that the related bias is small. The other operators offer similar mature solutions targeting all segments of the market and hence we expect their customers to be mostly similar. Even more, as the segregation at home and at work is rather different from segregation elsewhere in the activity space, those who have left out of the current study must show radically different behavior in order to substantially change the results above. We have no evidence that this is the case.
